# LLIN Evaluation in Uganda Project (LLINEUP) – Impact of long-lasting insecticidal nets with, and without, piperonyl butoxide on malaria indicators in Uganda: study protocol for a cluster-randomised trial

**DOI:** 10.1186/s13063-019-3382-8

**Published:** 2019-06-03

**Authors:** Sarah G. Staedke, Moses R. Kamya, Grant Dorsey, Catherine Maiteki-Sebuguzi, Samuel Gonahasa, Adoke Yeka, Amy Lynd, Jimmy Opigo, Janet Hemingway, Martin J. Donnelly

**Affiliations:** 1 London School of Hygiene & Tropical Medicine, Keppel Street, London, WC1E 7HT UK; 2 Makerere University College of Health Sciences, Infectious Diseases Research Collaboration, 2C Nakasero Hill Road, Kampala, Uganda; 3 Department of Medicine, University of California, San Francisco, San Francisco, CA 94110 USA; 4 National Malaria Control Division, Uganda Ministry of Health, Infectious Diseases Research Collaboration, 2C Nakasero Hill Road, Kampala, Uganda; 5 Infectious Diseases Research Collaboration, 2C Nakasero Hill Road, Kampala, Uganda; 6 Makerere University School of Public Health, Infectious Diseases Research Collaboration, 2C Nakasero Hill Road, Kampala, Uganda; 7 Liverpool School of Tropical Medicine, Pembroke Place, Liverpool, L3 5QA UK; 8 National Malaria Control Division, Uganda Ministry of Health, Kampala, Uganda

**Keywords:** Malaria, Long-lasting insecticidal nets, Piperonyl butoxide, Uganda, Cluster-randomised trial, Vector control, Insecticide resistance

## Abstract

**Background:**

Long-lasting insecticidal nets (LLINs) are a key malaria control intervention, but their effectiveness is threatened by resistance to pyrethroid insecticides. Some new LLINs combine pyrethroids with piperonyl butoxide (PBO), a synergist that can overcome P450-based metabolic resistance to pyrethroids in mosquitoes. In 2017–2018, the Ugandan Ministry of Health distributed LLINs with and without PBO through a national mass-distribution campaign, providing a unique opportunity to rigorously evaluate PBO LLINs across different epidemiological settings.

**Methods/design:**

Together with the Ministry of Health, we embedded a cluster-randomised trial to evaluate the impact of LLINs delivered in the 2017–2018 national campaign. A total of 104 clusters (health sub-districts) in Eastern and Western Uganda were involved, covering 48 of 121 (40%) districts. Using adaptive randomisation driven by the number of LLINs available, clusters were assigned to receive one of four types of LLINs, including two brands with PBO: 1) PermaNet 3.0 (*n* = 32) and 2) Olyset Plus (*n* = 20); and two without PBO: 3) PermaNet 2.0 (*n* = 37) and 4) Olyset Net (*n* = 15). We are conducting cross-sectional community surveys in 50 randomly selected households per cluster (5200 households per survey) and entomological surveillance for insecticide resistance in up to 10 randomly selected households enrolled in the community surveys per cluster (1040 households per survey) at baseline and 6, 12, and 18 months after LLIN distribution. Net durability and bio-efficacy will be assessed in 400 nets withdrawn from households with replacement at 12 months. The primary trial outcome is parasite prevalence as measured by microscopy in children aged 2–10 years in the follow-up surveys.

**Discussion:**

PBO LLINs are a promising new tool to reduce the impact of pyrethroid resistance on malaria control. The World Health Organization has issued a preliminary endorsement of PBO LLINs, but additional epidemiological evidence of the effect of PBO LLINs is urgently needed. The results of this innovative, large-scale trial embedded within a routine national distribution campaign will make an important contribution to the malaria control policy in Uganda and throughout Africa, where pyrethroid resistance in malaria vectors has increased dramatically. This model of evaluation could be a paradigm for future assessment of malaria control interventions.

**Trial registration:**

ISRCTN, ISRCTN17516395. Registered on 14 February 2017.

**World Health Organization Trial Registration Data Set:**

See Additional file 1.

**Electronic supplementary material:**

The online version of this article (10.1186/s13063-019-3382-8) contains supplementary material, which is available to authorized users.

## Background

Since 2000, coverage of malaria control interventions has expanded considerably across Africa, resulting in impressive reductions in malaria burden [1]. However, there is recent evidence that reductions in malaria burden have stalled in some countries [2]. In Uganda, progress on reducing malaria transmission has been slow, despite the scale-up of long-lasting insecticidal nets (LLINs), indoor residual spraying (IRS), and treatment of symptomatic malaria cases with artemisinin-based combination therapies, and control gains have been difficult to sustain [3–6]. Recent evidence that malaria incidence is rising in Uganda underscores the need to intensify malaria control efforts [2, 7].

LLINs reduce morbidity and mortality caused by malaria across a range of epidemiological settings and are the most widely used vector control tool in Africa [8, 9]. To achieve and maintain universal coverage with LLINs, the World Health Organization (WHO) recommends the distribution of one LLIN for every two individuals at risk of malaria through mass campaigns conducted every 3 years [10]. In Uganda, major efforts have been made to achieve universal coverage of LLINs [11], but LLIN coverage remains below target [2] and the impact of LLINs has been lower than expected in some areas of Uganda [5, 6]. Currently, all LLINs are treated with pyrethroid insecticides due to their favourable safety profile at low doses, repellent effects, rapid killing, and low cost. However, pyrethroid resistance has become widespread in Africa and presents a major threat to malaria control [12, 13]. Recently completed trials suggest that, even in areas of increased insecticide resistance, users of LLINs have lower prevalence and incidence of malaria than non-users, although these studies have not quantified the extent to which the community or mass effect may have been lost [14, 15].

Pyrethroid resistance is commonly mediated through two main mechanisms, including ‘knock-down resistance’ caused by target-site mutations in the receptor for pyrethroids (kdr) and metabolic resistance [13]. One of the most important metabolic resistance mechanisms is allelic and expression changes in cytochrome p450 enzymes that detoxify pyrethroids [16, 17]. To combat P450-mediated resistance, a new generation of LLINs has been developed which combines a pyrethroid insecticide with a synergist, piperonyl butoxide (PBO), which inhibits cytochrome P450s, and may thereby restore pyrethroid susceptibility [18]. Two brands of PBO nets have WHOPES (WHO Pesticide Evaluation Scheme) recommendations [19, 20]: PermaNet® 3.0 (Vestergaard Frandsen SA, Denmark) and Olyset® Plus (Sumitomo Chemical). Although both nets incorporate PBO, the characteristics of the PBO nets, and of the conventional non-PBO nets produced by both companies (PermaNet® 2.0 and Olyset® Net), vary in terms of insecticide used, concentrations, net weight, and construction [19, 20].

One epidemiological study of the effectiveness of LLINs with PBO has been conducted in Tanzania [21]. In this small, cluster-randomised, controlled trial which compared the impact of Olyset® Plus nets (with PBO) to Olyset® Nets (without PBO), parasite prevalence was significantly lower in the PBO LLIN arm at 9, 16, and 21 months after the intervention. Based on these results, the WHO has provided an interim endorsement of PBO LLINs, recommending that PBO LLINs be deployed in areas of intermediate-level pyrethroid resistance due at least in part to metabolic mechanisms [22]. However, the impact of LLINs with PBO is expected to vary according to transmission intensity, bioavailability and retention of PBO, and the mechanisms and level of pyrethroid resistance of local vectors. Additional evidence of the impact of combination LLINs (with PBO) is urgently needed.

In 2017–2018, the Ugandan Ministry of Health distributed LLINs with and without PBO through a national mass-distribution campaign, providing a unique opportunity to rigorously evaluate PBO LLINs across a variety of malaria transmission intensities, vector ecologies, and insecticide resistance patterns. In close collaboration with the Ministry of Health, we are conducting a cluster-randomised trial to evaluate the community-level impact of the LLINs delivered in the 2017–2018 national campaign at an unprecedented scale in Eastern and Western Uganda. This innovative, evaluation approach could be adopted for future assessments of malaria control interventions.

### Primary objective and hypothesis

We address the following research question: ‘Are combination LLINs (with PBO) more effective than conventional LLINs (without PBO) for malaria control in Uganda, an area with high-level P450-based pyrethroid resistance?’ We will test the hypothesis that parasite prevalence will be lower in intervention clusters (health sub-districts randomised to receive PBO nets) than in control clusters (health sub-districts randomised to conventional nets) and undertake a sub-group analysis stratified by region (Eastern and Western regions).

### Secondary objectives and hypotheses

In addition, the following secondary objectives will be addressed:

1. To evaluate the impact of PermaNet 3.0 (with PBO) compared with PermaNet 2.0 (without PBO) on parasite prevalence. We will test the hypothesis that parasite prevalence will be lower in intervention clusters (PermaNet 3.0 nets) than in control clusters (PermaNet 2.0 nets).
2. To evaluate the impact of Olyset Plus (with PBO) compared with Olyset Net (without PBO) on parasite prevalence. We will test the hypothesis that parasite prevalence will be lower in intervention clusters (Olyset Plus nets) than in control health clusters (Olyset Nets).
3. To determine the factors associated with the effectiveness of combination LLINs (with PBO) compared with conventional LLINs (without PBO), with a focus on the level of pyrethroid resistance. We will test the hypothesis that LLINs with PBO will be more effective than LLINs without PBO in settings with higher frequencies of P450-mediated pyrethroid resistance.
4. To assess net durability, bio-efficacy, survivorship, and use. We will conduct cross-sectional surveys to determine net survivorship and use, and to measure attrition, and will supplement these with laboratory assessments of net durability and bio-efficacy.

### Study design

**Fig. 1:**
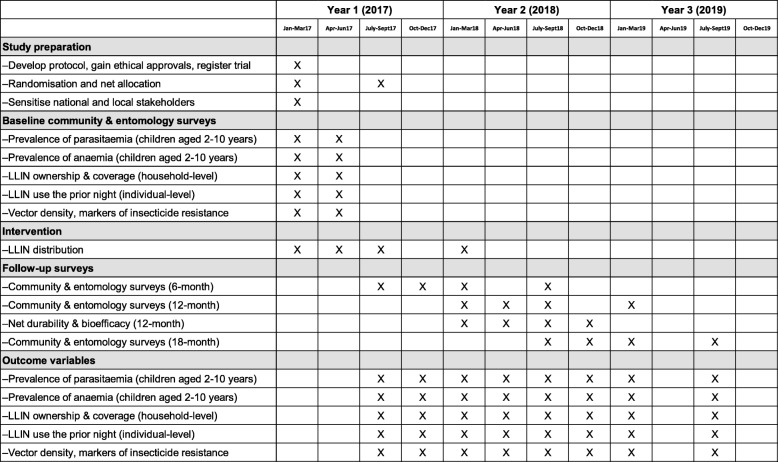
Trial timeline. LLIN long-lasting insecticidal net

The study includes 104 health sub-districts from 48 districts in Eastern and Western Uganda. The unit of randomisation is one health sub-district (cluster). Clusters were randomly assigned to receive one of four types of LLINs, including two LLINs with PBO: 1) PermaNet 3.0 (*n* = 32) and 2) Olyset Plus (*n* = 20); and two LLINs without PBO: 3) PermaNet 2.0 (*n* = 37) and 4) Olyset Net (*n* = 15). Prior to distribution of the nets, baseline surveys of community households and children aged 2–10 years, and entomological surveillance for insecticide resistance monitoring, were conducted (Fig. 1). LLINs were distributed within the study area by the Ministry of Health between March 2017 and March 2018. The evaluation includes follow-up community and entomology surveys at 6, 12, and 18 months post-distribution, plus assessment of net durability and bio-efficacy at 12 months. The primary outcome of the trial is parasite prevalence, measured by microscopy in children aged 2–10 years in the follow-up community surveys.

## Methods

### Study setting

**Fig. 2:**
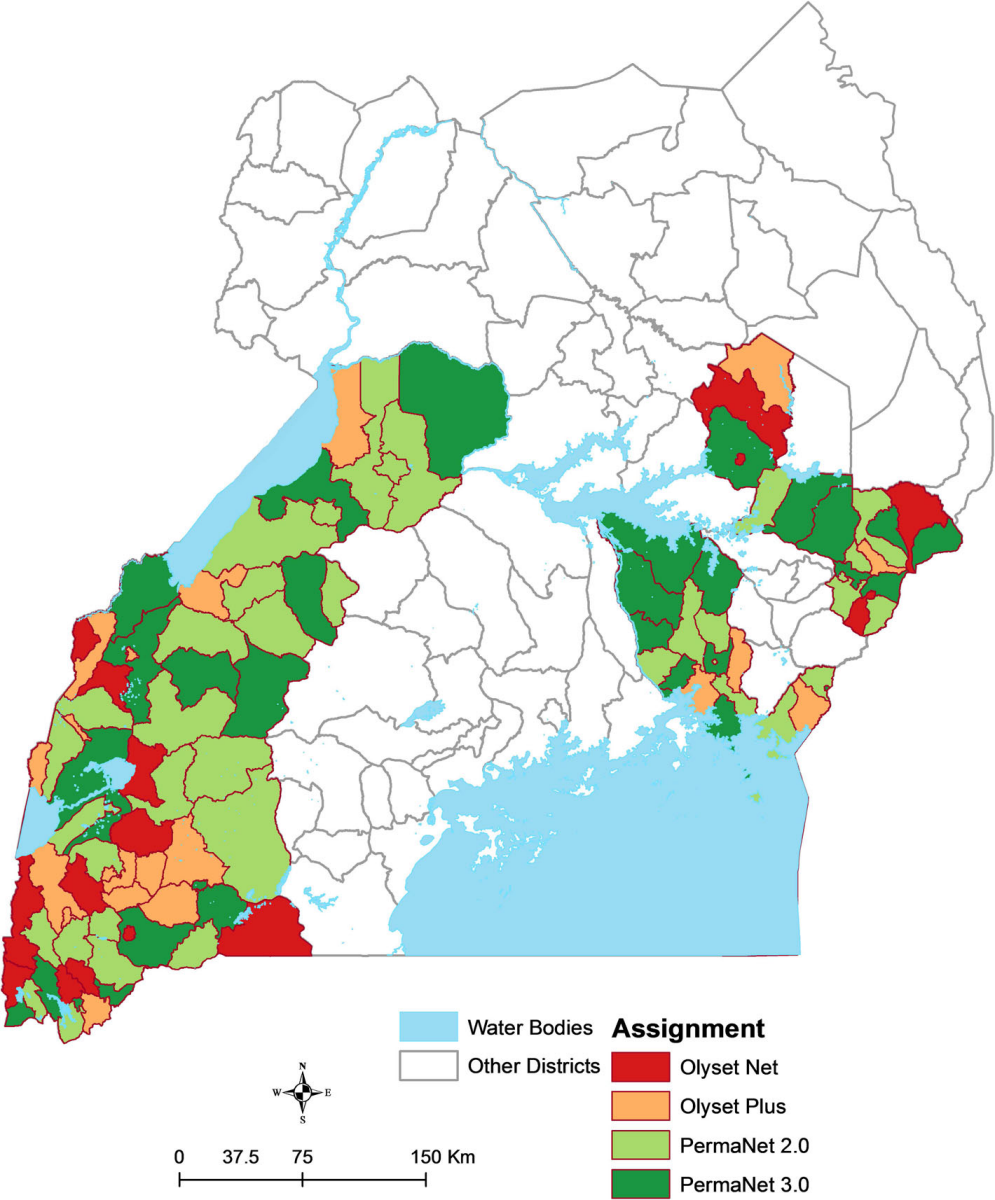
Map of the study area showing allocation of nets by cluster (health sub-district)

A total of 104 clusters in Eastern and Western Uganda were included in the study (Fig. 2). Areas scheduled to receive IRS with pirimiphos-methyl (Actellic) were excluded due to an interim WHO recommendation (since relaxed) that PBO nets should not be used in areas of Actellic spraying due to the possibility of antagonistic effects [22]. The study area represents five of the 10 geographic regions included in the Uganda Malaria Indicator Survey [23].

### Sensitisation

Prior to the study, project partners in the Ministry of Health and other key partners at the national and local level engaged with stakeholders at the district and community level in participating districts, including the District Health Officers and Malaria Focal Persons, local leaders, village health team (VHT) members, and other key opinion leaders. Study personnel used an information sheet to guide sensitisation discussions.

### Baseline community surveys

Cross-sectional surveys of community residents living in households randomly selected from each cluster were conducted from March to June 2017, prior to distribution of the LLINs. The community survey consisted of two components: 1) a household survey questionnaire administered to heads of households; and 2) a clinical survey of children aged 2–10 years.

#### Sampling frame

A two-stage cluster sampling procedure was applied using enumeration areas identified in the 2014 national census as the primary sampling unit [23, 24]. Ten enumeration areas within each of the 104 health sub-districts were randomly selected by the Uganda Bureau of Statistics (UBOS) using probability proportionate to size sampling. Households within each selected enumeration area were mapped and enumerated by the study team. A random sample of mapped households was selected from each enumeration area to generate a list of households to approach for recruitment.

#### Recruitment and enrolment

Households from the recruitment list were approached until five households from each enumeration area were enrolled (50 households per health sub-district, 5200 total). When a household was identified, study personnel briefly described the purpose of the study to the head of the household (or their designate) in the appropriate language. Households were included if: 1) at least one resident of the household was between 2 and 10 years of age; 2) at least one adult aged 18 years or older was present; 3) the adult was a usual resident who slept in the sampled household on the night before the survey; and 4) the adult resident agreed to provide informed consent for the household survey. Households were excluded if: 1) the dwelling was destroyed or not found; 2) the household was vacant; or 3) there was no adult resident at home on more than three occasions. Written consent to participate in the study was sought from an adult resident for all households fulfilling the selection criteria.

#### Household survey

Upon enrolment, a household survey questionnaire, adapted from prior cross-sectional community surveys conducted in Uganda including the national Malaria Indicator Survey [23, 25–27], was administered to heads of households or their designate using a hand-held tablet computer. Information was gathered on the characteristics of households and residents, proxy indicators of wealth including ownership of assets, and ownership and use of LLINs in the households.

#### Clinical survey

All children aged 2–10 years present in enrolled households were eligible to participate in the clinical survey. Sample collection for laboratory testing was performed in children identified from the household questionnaire if they met the following selection criteria: 1) were 2–10 years of age; 2) were usually resident and present in the sampled household on the night before the survey; 3) had provision of informed consent by a parent/guardian; and 4) had assent of child aged 8 years or older. If a child was not present of the day of the survey they were excluded. Finger-prick blood samples were taken from all eligible children for thick smear and for all eligible children 2–4 years of age for haemoglobin measurement. A filter paper blood sample was also obtained for future molecular testing.

#### Management of ill participants

Clinical survey participants who had a temperature ≥ 38.0 °C or who reported fever in the past 48 h had a rapid diagnostic test (RDT) performed (SD Bioline Malaria Ag P.f or P.f/Pan, Standard Diagnostics, Inc.). Participants with a positive RDT and no evidence of danger signs or severe malaria were treated with artemether-lumefantrine, which is the first-line treatment for uncomplicated malaria in Uganda. Participants with a positive RDT and evidence of danger signs of severe disease were referred for further evaluation and treatment. Any participant with other concerning clinical symptoms were also referred to an appropriate healthcare facility, following guidelines outlined in standard operating procedures.

### Baseline entomology surveys for insecticide resistance monitoring

Entomology surveys were carried out concurrently with the baseline community surveys to collect mosquito specimens for insecticide resistance monitoring. In each cluster, up to ten households were selected randomly from the list of 50 households enrolled into the community surveys. Study personnel re-visited households on the list of randomly selected households to carry out recruitment, and briefly described the purpose of the mosquito collections in the appropriate language to the head of household (or their designate) before proceeding with sampling. Mosquitoes were sampled from enrolled households in the mornings (from dawn until 10 am) using Prokopack aspirators to collect mosquitoes resting indoors on walls [28]. A brief questionnaire was also administered to gather information on household characteristics and the use of malaria prevention measures. Female anopheles mosquitoes were identified, and were stored at 4 °C in the regional field sites prior to shipment to Kampala and onto the Liverpool School of Tropical Medicine (LSTM) for molecular analysis.

### Assignment of interventions

#### Initial randomisation and net allocation

The randomisation was carried out by a member of the study team based outside of Uganda who was not directly involved in the field work. The total number of LLINs available for the four types of nets (corresponding to the four study arms) and the estimated number of LLINs needed for each health sub-district were established prior to the randomisation (Table 1). Given the restriction on the number of nets to be distributed in each study arm, adaptive randomisation was applied using the following steps: 1) A random number between 0 and 1 was generated for each cluster using STATA (StataCorp, Texas, USA). 2) Cumulative probability ranges were generated for each of the four types of net based on the targeted number of each individual type of net/targeted number of total nets. 3) The first cluster was assigned to their intervention based on which cumulative probability range the corresponding random number fell into. 4) Step 2 above was repeated after removing the number of targeted nets assigned to the cluster for the corresponding type of net and the total number of nets. 5) The next cluster was assigned to their intervention based on which revised cumulative probability range the corresponding random number fell into. 6) This process was repeated until all clusters were allocated to an intervention. The process was stratified by region, with 66 clusters in the Western region and 38 clusters in the Eastern region since although insecticide resistance is very well characterised in Eastern Uganda, little is known about patterns in the West [17, 29–31]. Areas scheduled to receive IRS with pirimiphos-methyl (Actellic) were excluded [22]. Given the nature of the trial, allocation of the LLINs was not blinded.

**Table 1:** Revised study interventions, target number of nets, and final number of clusters allocated

Type of LLIN	Targeted total number of nets for distribution		Cumulative number of nets allocated		Number of clusters (health sub-districts) allocated				
	Original randomisation	Final randomisation	Original randomisation	Final randomisation	Waves 2 and 3	Wave 4	Western region	Eastern region	Total
PermaNet 2.0	3,217,360	3,743,320	3,220,280	3,830,280	16	21	24	13	37
PermaNet 3.0 (contains PBO)	4,100,000	3,570,640	4,101,840	3,515,520	18	14	16	16	32
Olyset Net	650,000	1,244,400	651,240	1,239,640	4	11	11	4	15
Olyset Plus (contains PBO)	1,000,000	1,601,560	994,000	1,574,480	6	14	15	5	20
Total	8,967,360	10,159,920	8,967,360	10,159,920	44	60	66	38	104

*LLIN* long-lasting insecticidal net, *PBO* piperonyl butoxide

#### LLIN distribution

The LLINs were distributed by the Ministry of Health and supporting partners according to national guidelines [32]. Household-level registration data were collected door-to-door in each village to determine the number of nets needed [32]. The LLINs were imported into Uganda and stored in warehouses in Kampala. The Ministry of Health was responsible for issuing the LLINs according to the net allocation list. Nets were taken out of the Kampala warehouses and placed onto trucks for transport to sub-county stores. Using the household registration data, and in accordance with WHO recommendations, an allocation formula was applied (total number of people in the household, divided by 2, and rounded up in the case of an uneven number of household members) to determine the number of nets each household was to receive [32]. Community members were mobilised and informed in advance of the distribution.

#### Revised randomisation and net allocation

Distribution of LLINs in the first 44 clusters was completed by the Ministry of Health as scheduled, with nets distributed in 24 PBO and 20 non-PBO clusters (Table 1). However, the number of nets distributed initially was greater than estimated, resulting in a deficit of nets available for the final round of distribution within the study area. After determining the total number of nets available, the 60 remaining clusters were re-randomised, using the same process of adaptive randomisation described above, such that the number of clusters assigned to the two study arms (PBO versus non-PBO) balanced, with 52 clusters assigned to each of the two study arms in a 1:1 ratio (Table 1).

### Data collection

#### Follow-up cross-sectional surveys

To evaluate the impact of the LLINs, cross-sectional surveys of community residents will be conducted at 6, 12, and 18 months post-distribution, following the same methods as described above for the baseline surveys. In brief, the follow-up community surveys will consist of a household survey questionnaire administered to heads of households, and a clinical survey of children aged 2–10 years. Households will be randomly selected from each of the 104 clusters using two-staged cluster sampling until 50 households are enrolled per cluster (total 5200 households). The household questionnaire used for the baseline surveys will be adapted for the follow-up surveys to include specific questions about the LLINs distributed by the Ministry of Health through the national campaign. Random selection of households for screening and enrolment will be repeated with replacement for each cross-sectional survey. All children aged 2–10 years from enrolled households who are present will be eligible to participate in the clinical survey. The primary outcome of the community surveys will be malaria parasite prevalence as measured by microscopy. Entomological surveillance for insecticide resistance will also be conducted concurrently with each community survey, aiming to collect mosquitoes from 10 randomly selected households per clusters as described above.

#### Phenotypic monitoring

We intend to conduct resistance phenotyping on mosquito samples collected from a sub-set of households located in 12 sentinel site clusters, which will be purposely selected based on the likelihood of collecting large numbers of mosquitoes, supported by baseline entomology data, and by proximity to the regional entomology laboratories where the assays will be performed. Six clusters will be selected from each region (East and West), aiming to balance the net allocation assignments in each region. In each cluster, approximately 10–15 households will be selected or approximately 120–180 households in total, per survey. The same procedures described above will be followed for recruitment, screening, consenting, surveying, and mosquito collection, with one exception: households may be visited more than once for mosquito collections. The live females collected will be tested for susceptibility to the active ingredients used on the distributed LLINs (Olyset, permethrin; PermaNet, deltamethrin) and in combination (permethrin + PBO and deltamethrin + PBO). Susceptibility assays will be conducted in line with WHO guidelines, and we will combine standard WHO tube tests with intensity assays where indicated. If the number of adult females obtained from resting collections is too small they will be supplemented by 3- to 5-day old females raised from larval collections. We propose to test the susceptibility of the three major vectors (*An. gambiae, An. arabiensis* and *An. funestus*), but it is possible that too few mosquitoes will be collected to make an accurate assessment of population susceptibility.

#### LLIN durability and bio-efficacy

Durability and bio-efficacy of the LLINs will be assessed 1 year after the distribution of nets, concurrently with the 12-month cross-sectional survey. We will quantify net durability and bioavailability of insecticides using standard WHO methodologies. To assess LLIN durability (the number, location, size, and type of holes in each net), a sub-set of nets distributed during the 2016–2017 universal LLIN campaign will be withdrawn (and replaced) from households during the 12-month cross-sectional survey. We propose to sample 400 nets (100 per study arm) from randomly selected households using the list of households selected for the cross-sectional community surveys. During the cross-sectional survey, availability of nets distributed during the 2016–2017 universal LLIN campaign will be assessed in selected households. If nets are available in the household, one net per household will be withdrawn (and replaced) for the durability assessment. We will quantify durability of the net using the methodology developed by the WHO Vector Control Working Group [33].

To assess LLIN bio-efficacy (the degree of knock-down, mortality, or inhibition of blood-feeding induced in susceptible mosquitoes, as determined by cone bioassays), the sub-set of 400 nets will also undergo bio-efficacy assessments. We will use a standard laboratory strain of *An. gambiae* from Kisumu, Western Kenya, which is fully susceptible to both permethrin and deltamethrin in WHO discriminant dose tests. In brief, five unfed 3- to 5-day-old mosquitoes will be exposed for 3 min to a net sample taken from the top of each net. The rationale being that this is the first point of contact for a mosquito taking a blood meal and ensures that we sample the dual treated surface on both types of PBO net [34, 35]. Knock-down will be recorded 60 min post-exposure and mortality after 24 h. Two net samples will be used per net and five cone tests will be conducted per sample together with appropriate negative controls. For a subset of 20 nets of each type we will use the same protocol but with a Ugandan pyrethroid resistant strain which exhibits P450-mediated resistance to test for the durability of PBO treatment on the dual-treatment nets.

### Laboratory procedures

#### Microscopy

Thick blood smears will be made by placing a drop of blood in the middle of a barcoded slide. Slides will be dried and kept in the field for no longer than 7 days to avoid auto-fixation. While in the field, slides will be stored in a cool environment and protected from excess heat and sunlight. Blood samples will be transported periodically to the Infectious Diseases Research Collaboration (IDRC) Molecular Research Laboratory (MOLAB) in Kampala for reading. At the MOLAB, thick blood smears will be stained with 2% Giemsa for 30 min and then evaluated for the presence of parasitaemia (asexual forms only). A thick blood smear will be considered negative when the examination at 100× high-power fields does not reveal asexual parasites. For quality control, all slides will be read by a second microscopist. If there is a discrepancy between the first and second reads (defined as any of the following: 1) a positive versus negative for asexual parasites, 2) parasite density differing by ≥ 25%, and 3) positive versus negative for gametocytes), a third microscopist will review the slide.

#### Haemoglobin measurement

Haemoglobin analysis will be carried out in eligible children 2–4 years of age on site using a drop of blood collected from a finger-prick using a battery-operated portable analyser (HemoCue, Anglom, Sweden). Any participant who is found to have severe anaemia (haemoglobin < 7.0 g/dL) requiring treatment will be referred to an appropriate healthcare facility for further management.

#### Filter paper samples

Blood spots will be collected onto filter paper to store for future serologic and/or molecular studies which will be performed only for research purposes and will have no impact on the clinical management of study participants. Filter paper (Whatman no 1, Whatman 3MM; Whatman, Maidstone, UK) will be pre-cut into individual squares, stapled to a thick card which will serve as its cover, and will be labelled with a barcode. Blood spots will be collected onto the filter paper in volumes of approximately 25-μl aliquots per blood spot (four blood spots per sample). Filter paper samples will be allowed to dry at ambient temperature and relative humidity, transported from the field in a zip lock bag, and will be placed into a stock card filter paper box for final storage with a desiccant.

#### Rapid diagnostic tests

RDTs (SD Bioline Malaria Ag P.f or P.f/Pan, Standard Diagnostics, Inc.) will be performed in the field on cross-sectional survey participants who are found to have a temperature ≥ 38.0 °C or who report fever in the past 48 h. RDTs will be performed according to the directions provided for the specific tests, using the blood transfer device and reagent provided by the manufacturer. Tests will be performed by study personnel, and results will be available within 15 min. The results of the RDT will be provided to the participant’s caregiver verbally and will be recorded on the appropriate case record form. Participants who test positive for malaria will be provided with a full course of anti-malarial treatment and will also be counselled to go to the nearest healthcare facility immediately if their condition worsens.

#### Insecticide resistance monitoring

We plan to use a combination of single nucleotide polymorphisms in the P450 *Cyp4j5* and the esterase *Coeae1d* which were reproducibly associated with pyrethroid resistance in multiple field collections from Uganda and Kenya, and which together with the *Vgsc*-1014S (kdr) mutation explained around 20% of variation in pyrethroid resistance [17]. These will be supplemented by Ugandan-specific resistance markers identified through the *Anopheles gambiae* 1000 genomes project (Ag1000G), and allied projects [30, 36]. Female anopheles mosquitoes collected from the 104 clusters during the entomology surveys will be identified and will be stored and refrigerated in the field sites prior to shipment to Kampala and onto LSTM for further analysis.

### Data management

All data will be collected by survey teams using hand-held tablet computers. Prior to conducting the surveys, information from the questionnaires and fields for entering results of laboratory testing will be programmed into the tablet computers. Programming will include range checks, structure checks, and internal consistency checks. Before leaving the household, an inventory will be made of the completed questionnaires and blood samples collected; both will be checked to make sure they are labelled correctly. The completed questionnaires will be checked for mistakes and completeness. Data from these devices will be transferred at the end of every day to our data core facilities in Kampala and stored on a secure server. The data file will be kept on a separate network so that only authorized survey staff will have access to the data during collection and the processing phase. The file with data from the questionnaires will be merged with results from reading the malaria slides at the laboratory, using the unique bar codes. All filter paper samples and blood slides will be returned to the IDRC offices in Kampala.

Laboratory data, including results of microscopy, will be recorded by study personnel on standardized data forms. Data entered onto paper record forms will be entered into a computerised database (Microsoft Access) by a data entry clerk and will be double-entered to verify accuracy. An audit trail of the date and time of data entry, and a record of any changes made, will be kept in compliance with Good Clinical Practice (GCP). All study personnel will be trained in the project objectives, methods of effective communication with study participants, collection of high-quality data, and principles of ethical research practice. Study personnel members will receive additional training specific to the tasks they will perform within the project including interviewing techniques, administration of surveys, completing questionnaires, and use of tablet devices. Standard operating procedures (SOPs) will be written for all project activities, and booklets of all relevant documents provided to each member of the project team.

Records for this study will be maintained and stored in compliance with the principles of GCP and regulatory and institutional requirements, and in compliance with the requirements for the protection of confidentiality of participants.

### Sample size calculations

#### Community surveys

The study sample size (number of clusters and allocation of interventions) was set by the number of nets available for the four different types of study nets and the estimated number of LLINs required per cluster. We will sample all eligible children aged 2–10 years from 50 households in the 104 clusters in each round of surveys, resulting in an estimated total of 10,400 children from 5200 households per survey. Assuming a parasite prevalence of 40% in the control arm [25], and coefficient of variation between clusters of 0.3 (derived from the ongoing trial in Tanzania [21]), we will have 80% power (two-sided significance level of 0.05) to detect a relative reduction in parasite prevalence of at least 17% (prevalence ratio of 0.83).

#### Entomology surveys

We aim to collect 30–50 mosquitoes per cluster. The sample sizes were chosen based on the expected frequency (50 to 60%) of the known resistance-associated P450 variant (*Cyp4j5*) in Eastern Uganda [17]. Therefore, a relatively small sample size is required for an accurate estimate of resistance marker frequency. However, we are aware that resistance is a highly dynamic system and that we may need to increase the number of mosquitoes analysed in certain clusters if resistance marker frequencies change dramatically.

### Analytical issues

#### Primary and secondary outcomes

The primary and secondary outcomes are outlined in Table 2.

**Table 2:** Outcome measure definitions

	Indicator definition
Community survey	
Prevalence of parasitaemia	Proportion of thick blood smears that are positive for asexual parasites
Prevalence of anaemia	Proportion of haemoglobin measurements categorized as anaemia (< 10.0 g/dL)
LLIN coverage	Proportion of households owning at least one net
	Proportion of households owning at least one LLIN
	Average number of nets per household
	Average number of LLINs per household
	Proportion of households with one LLIN for every two residents
	Proportion of children who slept under an LLIN the prior night
	Proportion of pregnant women who slept under an LLIN the prior night
Insecticide resistance	
Phenotyping	Prevalence of phenotypic insecticide resistance
Genotyping	Prevalence of molecular markers associated with insecticide resistance
LLINs	
Survivorship	Proportion of households owning at least one 2016–2017 campaign net
	Proportion of households using at least one 2016–2017 campaign net the prior night
Durability	Proportionate Hole Index (PHI):
	1) good, 2) serviceable (repairable), or 3) in need of replacement
Bio-efficacy	Knock-down rate at 60 min of mosquitoes tested
	Mortality rate of mosquitoes tested

*LLIN* long-lasting insecticidal net

#### Statistical methods

All data will be analysed on the basis of intention-to-treat and per-protocol analyses. Intention-to-treat analyses will include the assigned treatment arms and results from all clusters. For per-protocol analyses, clusters will be grouped by treatment arm according to the type of LLINs actually received in the national campaign. For clusters with mixed LLIN distribution, the number of nets from the dominant type received (numerator) will be divided by the total number of the four study net types received in that cluster, excluding non-study nets (denominator). To be included in the per-protocol analyses, the proportion of the dominant net received in the cluster must be > 75% out of the four study net types. Clusters in which the dominant net type received is ≤ 75% will be excluded from the per-protocol analyses. The primary outcome will be the prevalence of asexual parasitaemia from the cross-sectional surveys. An individual-level approach to the analysis will be used due to the large number of clusters per arm. For comparison of the primary outcome between treatment arms, generalized linear Poisson models with a log-link function will be used to allow for within-cluster correlations adjusting for baseline cluster-level parasite prevalence. The effect of the intervention will be quantified by calculation of a prevalence ratio. Separate analyses will be performed using data from the post-distribution cross-sectional surveys (performed 6, 12, and 18 months after the delivery of the nets).

#### Sub-group analyses

Stratified sub-group analyses will be performed at the level of the region (Western or Eastern) and net manufacturer (PermaNet or Olyset). Spatial auto-correlation in insecticide resistance will also be explored to interpolate resistance patterns collected at the household level.

### Monitoring

Given the nature of this trial, in which the intervention (LLINs) is delivered as a national programme by the Uganda Ministry of Health, no data and safety monitoring committee was established, and we have no plans for an interim analysis. Moreover, as the investigators are responsible only for the evaluation, we have no plans for monitoring adverse events. Internal audits of the trial are being conducted for each round of surveys by the IDRC’s regulatory department.

## Ethical issues

### Research ethics approval

The trial has been approved by the Ugandan National Council for Science and Technology (UNCST; ref. HS 2176), Makerere University School of Medicine Research & Ethics Committee (SOMREC; 2016–133), London School of Hygiene and Tropical Medicine Ethics Committee (LSHTM; ref. 12019), and the Liverpool School of Tropical Medicine (LSTM; ref. 16–072), which sponsored the study.

### Protocol amendments

Approval of the original study protocol (V2.0, 1 February 2017) was obtained from all of our Institutional Review Boards (IRBs) on 13 March 2017. The protocol was subsequently amended (Version 3.0, 15 August 2017), and was fully approved on 1 November 2017. For any future protocol amendments, investigators will be notified, IRB approval will be sought, and the clinical trial registry will be updated. For the SPIRIT 2013 Checklist of recommended items to include in an interventional trial protocol, see Additional file 2.

### Informed consent

Approval from local leaders will be sought before beginning activities in the project area. Written informed consent to participate in the study will be obtained by the head of household (or their designate) for all households participating in the community and entomology surveys. Written consent to participate in the clinical survey will be obtained from parents or guardians of all children. In all cases involving participation of children aged 8 years or older, written assent will also be obtained from the child. All informed consent discussions will be conducted in the appropriate local language, and a translator will be used if necessary. Information sheets and consent forms will be available in English and appropriate local languages, describing the purpose of the project and the procedures to be followed, and the risks and benefits of participation. During the consent discussions, each section of the consent form will be read exactly as it is written either by study personnel or by the translator, and then further explained to the respondent (participant or parent/guardian) if necessary. The translator will also assist with the discussion and assessment of comprehension. All participants and parents/guardians will be informed that participation in the study is completely voluntary and that they may withdraw from the study at any time. If the person asked to provide consent is unable to read or write, their fingerprint will substitute for a signature, and a signature from a witness to the informed consent procedures will be obtained. Written consent for future use of biological specimens will also be obtained for the community surveys.

### Confidentiality

Records for this study will be maintained and stored in compliance with the principles of GCP and regulatory and institutional requirements, and in compliance with the requirements for the protection of confidentiality of participants. Only study personnel will have access to these records. All forms with participant names will be kept in a locked cabinet when not in use. Participants will be identified by their study ID number, and participant names will not be included in databases used for analysis. Data will be stored for at least 10 years. Anonymized data collected in this study may also be shared with other investigators and/or placed into the public domain via a data repository.

### Ancillary and post-trial care

Given the nature of this trial, in which the intervention (LLINs) is delivered as a national programme by the Uganda Ministry of Health and the evaluation includes assessments of community members at a single point in time, there are no plans to provide ancillary or post-trial care.

### Dissemination policy

The final 18-month cross-sectional survey will be conducted in September 2019. Laboratory testing, data analyses, and preparation of the final manuscript are expected to take until the end of 2019. Peer-review and publication of the trial results are anticipated in the first quarter of 2020. We plan to disseminate the findings widely, sharing with the sponsor and key stakeholders, including the Uganda Ministry of Health and the National Malaria Control Division, the World Health Organization’s Vector Control Advisory Group, Innovative Vector Control Consortium, President’s Malaria Initiative, and Global Fund, among others. The results will also be presented at national and international conferences. Authorship for the Long-Lasting Insecticidal Net Evaluation in Uganda Project (LLINEUP) manuscripts was decided in advance by the core investigators; no professional writers will be used. Access to the full protocol, participant-level dataset, and statistical code will be available upon reasonable request.

## Trial status

The baseline community and entomology surveys were carried out from March to June 2017. LLINs were distributed in the study area between March 2017 and March 2018. The first round of follow-up surveys (6 months) started in September 2017 and the final round of surveys (18 months) are scheduled to complete in September 2019. We are seeking funding for an additional round of surveys, which would be conducted at 24–30 months following distribution of the LLINs.

## Discussion

The WHO has issued a preliminary endorsement of PBO LLINs based on the results of a single trial conducted in Tanzania, but additional evidence of the effect of PBO LLINs is urgently needed. Uganda, with its persistently high burden of malaria and widespread pyrethroid resistance, is an optimal setting to undertake this evaluation. We have leveraged resources by applying a cluster-randomised design to a national LLIN distribution campaign aiming to achieve universal coverage of LLINs in Uganda. Although this study design, linking the evaluation to the national LLIN campaign, presents substantial opportunities, it also presents a few limitations. Because the nets were procured in advance of establishing the trial, the type and number of nets were already fixed. Thus, we were required to use adaptive randomisation to accommodate for the differences in net availability. The shortage of nets available for the final round of distribution in the study area forced the Ministry of Health to source additional nets; as a result, the research team had to re-randomise the final 60 clusters. Moreover, the Ministry of Health and partners, not the research team, were responsible for distributing the nets. Although we worked closely with the Ministry of Health to ensure that the LLINs were allocated according to the randomisation list, there is a chance that the net distribution was imperfect. Despite these challenges, this innovative trial design demonstrates how policy makers, programme implementers, and researchers can work together to generate robust scientific evidence on the impact of malaria control interventions delivered at a national level.

## Roles and responsibilities

### Investigators listed on the protocol

Principal investigator (PI): Prof. Janet Hemingway, CBE, FRS, DSc, PhD, BSc, FRCP (Hon), FMedSci, FRES (Hon), (foreign associate National Academy of Sciences USA), FAAM, Hon FFPH; Director, Liverpool School of Tropical Medicine, UK.

Uganda Co-PI: Prof. Moses Kamya, MBChB, MPH, PhD; Professor and Dean, Makerere University College of Health Sciences, Kampala, Uganda; Executive Director, Infectious Disease Research Collaboration, Kampala, Uganda.

Uganda Co-PI: Prof. Sarah Staedke, MD, PhD, DTM&H; Professor of Malaria & Global Health, London School of Hygiene & Tropical Medicine, UK; Infectious Disease Research Collaboration, Kampala, Uganda.

Co-investigator: Prof. Grant Dorsey, MD, PhD; Professor, Department of Medicine, University of California, San Francisco, USA; Infectious Disease Research Collaboration, Kampala, Uganda.

Co-investigator: Prof. Martin Donnelly, MA, MSc, PhD, FRES; Professor of Evolutionary Genetics, Liverpool School of Tropical Medicine, UK.

Co-investigator: Dr. Yeka Adoke, MBChB, MPH, PhD; Lecturer, Makerere University School of Public Health, Kampala, Uganda; Infectious Disease Research Collaboration, Kampala, Uganda.

Co-investigator: Prof. Hilary Ranson, BSc, MSc, PhD; Professor of Medical Entomology, Liverpool School of Tropical Medicine, UK.

Co-investigator: Prof. Anthony Mbonye, MBChB, MA, MA, PhD; Commissioner of Health Services, Uganda Ministry of Health, Kampala, Uganda; Associate Professor of Public Health, Makerere University, Kampala, Uganda.

Co-investigator: Dr. Jimmy Opigo, MBChB; Manager, National Malaria Control Programme

Uganda Ministry of Health, Kampala, Uganda.

Co-investigator: Dr. Catherine Maiteki-Sebuguzi, MBChB, MSc; National Malaria Control Program, Uganda Ministry of Health, Kampala, Uganda; Infectious Disease Research Collaboration, Kampala, Uganda.

### Trial sponsor

This trial is sponsored by the Liverpool School of Tropical Medicine (LSTM); Research Protocol (#16–072).

### Role of sponsor and funders

Members of the LSTM (sponsor), including JH and MJD, contributed to the design of the study and drafting of this manuscript. They also support the ongoing collection, management, analysis, and interpretation of data. The ultimate authority over these activities is held jointly by the core investigative team, consisting of JH and MJD (LSTM), SGS (London School of Hygiene & Tropical Medicine), MRK (Makerere University/Infectious Diseases Research Collaboration), and GD (University of California, San Francisco). The funders played no role in these activities.

### Trial coordination

The project is coordinated by the core investigative team, and field activities are carried out in Uganda by the Infectious Diseases Research Collaboration led by a local study coordinator. Daily management of the trial is overseen by SGS supported by MRK; data management is overseen by GD. No steering committee or endpoint adjudication committee have been appointed for this trial.

## Additional files

Additional file 1: World Health Organization trial registration dataset. (PDF 118 kb)

Additional file 2: SPIRIT 2013 Checklist: standard protocol items: recommendations for interventional trials. (DOC 123 kb)

## Abbreviations

GCP: Good Clinical Practice
IDRC: Infectious Diseases Research Collaboration
IRS: Indoor residual spraying
LLIN: Long-lasting insecticidal net
LLINEUP: Long-Lasting Insecticidal Net Evaluation in Uganda Project
LSHTM: London School of Hygiene and Tropical Medicine
LSTM: Liverpool School of Tropical Medicine
PBO: Piperonyl butoxide
RDT: Rapid diagnostic test
SOP: Standard operating procedure
UBOS: Uganda Bureau of Statistics
WHO: World Health Organization
