# Supplementary material for: LLIN Evaluation in Uganda Project (LLINEUP) – Impact of long-lasting insecticidal nets with, and without, piperonyl butoxide on malaria indicators in Uganda: study protocol for a cluster-randomised trial
Source: Trials. 2019 Jun 3;20:321. doi: 10.1186/s13063-019-3382-8 (PMC6547536; doi:10.1186/s13063-019-3382-8)
Supplement: Supplementary file 1 — World Health Organization trial registration dataset. (PDF 118 kb) [file 13063_2019_3382_MOESM1_ESM.pdf]

Additional File 2: World Health Organization Trial Registration Data Set

LLIN Evaluation in Uganda Project (LLINEUP) – Impact of long-lasting insecticidal nets with, and without, piperonyl butoxide on malaria indicators in Uganda: study protocol for a cluster-randomised trial

Primary registry and trial identifying number	ISRCTN, ISRCTN17516395. https://doi.org/10.1186/ISRCTN17516395
Date of registration in primary registry	Registered 14 February 2017
Secondary identifying numbers	N/A
Source(s) of monetary or material support	Against Malaria Foundation, Department for International Development and the Innovative Vector Control Consortium
Primary sponsor	Liverpool School of Tropical Medicine
Secondary sponsor(s)	N/A
Contact for public queries	Prof Martin Donnelly; martin.donnelly@lstmed.ac.uk<br><br>Liverpool School of Tropical Medicine<br>Pembroke Place<br>Liverpool<br>L35QA<br>United Kingdom<br>+44 151 705 3296
Contact for scientific queries	Prof Martin Donnelly; as above
Public title	LLIN Evaluation in Uganda Project (LLINEUP) – Impact of long-lasting insecticidal nets with, and without, piperonyl butoxide on malaria indicators in Uganda: study protocol for a cluster-randomised trial
Scientific title	Uganda PBO Net Study: Impact of long-lasting insecticide treated bednets with and without piperonyl butoxide (PBO) on malaria indicators in Uganda: a cluster-randomised trial
Acronym	LLINEUP
Countries of recruitment	Uganda
Health condition(s) or problem(s) studied	Malaria
Intervention(s)	Long-lasting insecticidal nets (LLINs) with, and without, piperonyl butoxide (PBO)
Key inclusion and exclusion criteria –<br><br>For the community surveys	For household, inclusion criteria are:<br>1 At least one household resident between 2-10 years of age present (with an adult caregiver willing to provide informed consent for the clinical survey)<br>2 At least one adult aged 18 years or older present<br>3 Adult is a usual resident who slept in the sampled household on the night before the survey<br>4 Agreement of the adult resident to provide informed consent for the household survey

	For households, exclusion criteria are:<br>1 Dwelling destroyed or not found<br>2 Household vacant<br>3 No adult resident home on more than 3 occasions<br><br>For children, inclusion criteria are:<br>1 Child aged 2-10 years<br>2 Usual resident who was present in the sampled household on the night before the survey<br>3 Agreement of parent/guardian to provide informed consent<br>4 Agreement of child aged 8 years or older to provide assent<br><br>For children, exclusion criterion is:<br>1 Child not home on day of survey
Study type	• Interventional<br>• Cluster-randomised trial<br>• Not blinded
Date of first enrolment	16 March 2017
Target sample size	For each community survey, 50 randomly selected households will be enrolled from all 104 health sub-districts (clusters); all eligible children aged 2-10 years from participating households will be recruited. Target number per survey: 5200 households, ~10,000 children
Recruitment status	Ongoing
Primary outcome(s)	Parasite prevalence (defined as the proportion of thick blood smears that are positive for asexual parasites) in children aged 2-10 years is assessed using cross-sectional surveys at baseline (prior to distribution of the nets) and up to 3 times after nets are distributed, 6, 12 and 18-24 months after distribution.
Key secondary outcomes	1 Prevalence of anaemia and mean haemoglobin in children aged 2-10 years. Anaemia will be defined as a haemoglobin concentration (g/dl) less than 11 in children 24-59 months and less than 11.5 in child 5-10 years. Haemoglobin concentration will be measured on site using a drop of blood collected from a finger-prick. The test will be conducted during the cross-sectional surveys using a battery-operated portable HemoCue analyzer (HemoCue, Anglom, Sweden) at baseline (prior to distribution of the nets) and up to 3 times after nets are distributed, 6, 12 and 18-24 months after distribution.<br><br>2 Frequency of molecular markers associated with insecticide resistance in the primary malaria vector will be conducted on DNA extracted from mosquitoes collected in each of the study clusters during the cross-sectional surveys and will utilise PCR and rtPCR approaches at baseline (prior to distribution of the nets) and up to 3 times after nets are distributed, 6, 12 and 18-24 months after distribution.

	3 Prevalence of phenotypic insecticide resistance in 12 study clusters will be assessed using standard World Health Organization (WHO) tests of insecticide resistance performed during the cross-sectional surveys at baseline (prior to distribution of the nets) and up to 3 times after nets are distributed, 6, 12 and 18-24 months after distribution.<br><br>4 LLIN survivorship, durability and bio-efficacy will be assessed during a cross-sectional survey conducted 12 months after distribution. The tests will follow WHO guidelines: survivorship defined as the presence or absence of a LLIN in a survey household; durability is defined based on the number and area of holes in the LLIN; bio-efficacy is defined based the proportion of known susceptible mosquitoes surviving exposure to the LLIN netting.
